# Preliminary tide gauge evidence for equilibrium and non-equilibrium pole tide variability after the 2015 Chandler Wobble amplitude reduction

**DOI:** 10.1186/s40623-026-02461-4

**Published:** 2026-05-19

**Authors:** Taehwan Jeon, Ki-Weon Seo, Kookhyoun Youm, Jooyoung Eom, Jianli Chen, Clark R. Wilson

**Affiliations:** 1 Center for Educational Research, Seoul National University, Seoul, 08826 Republic of Korea; 2 Department of Earth Science Education, Seoul National University, Seoul, 08826 Republic of Korea; 3 Division of Glacier and Earth Sciences, Korea Polar Research Institute, Incheon, 21990 Republic of Korea; 4 Department of Earth Science Education, Kyungpook National University, Daegu, 41566 Republic of Korea; 5 State Key Laboratory of Climate Reseilience for Coastal Cities, Department of Land Surveying and Geo-Informatics, The Hong Kong Polytechnic University, Hong Kong, China; 6 Research Institute for Land and Space, The Hong Kong Polytechnic University, Hong Kong, China; 7 The Hong Kong Polytechnic University Shenzhen Research Institute, Shenzhen, China; 8 Center for Space Research, University of Texas at Austin, Austin, TX 78759 USA; 9 Department of Earth and Planetary Sciences, Jackson School of Geosciences, University of Texas at Austin, Austin, TX 78712 USA

**Keywords:** Non-equilibrium pole tide, Chandler Wobble, Tide gauge measurement

## Abstract

The pole tide has been defined as the oceanic response to polar motion, with a component due to the Chandler Wobble (CW), at a period near 14 months. The magnitude of the CW was greatly reduced after about 2015, and a corresponding global attenuation of the ocean pole tide amplitude is expected. We analyzed global tide gauge measurements using empirical orthogonal functions (EOF) methods to separate temporal and spatial patterns of regional pole tide signals. Our results show that tide gauge arrays in Japan and along the east coast of North America exhibit 14-month pole tide signals whose temporal variability is broadly consistent with the CW amplitude reduction after 2015. At stations in both regions, EOF-based mean pole tide amplitudes since 1980 fall in the range of approximately 0.5–1 cm, representing a more coherent signal than direct spectral estimates from individual stations. These amplitudes exceed the expected equilibrium response (less than 0.5 cm), suggesting that they reflect a mixture of equilibrium and moderate non-equilibrium contributions. Tide gauges in the North Sea and Baltic Sea show no detectable amplitude decrease after ~ 2015, consistent with previous studies documenting strong non-equilibrium pole tide variability in these regions. Together with the partial agreement with equilibrium expectations in Japan and along the North American east coast, these results suggest that regional pole tide signals likely reflect a combination of equilibrium response and additional forcing, potentially including long-period atmospheric variability.

**Graphical abstract:**

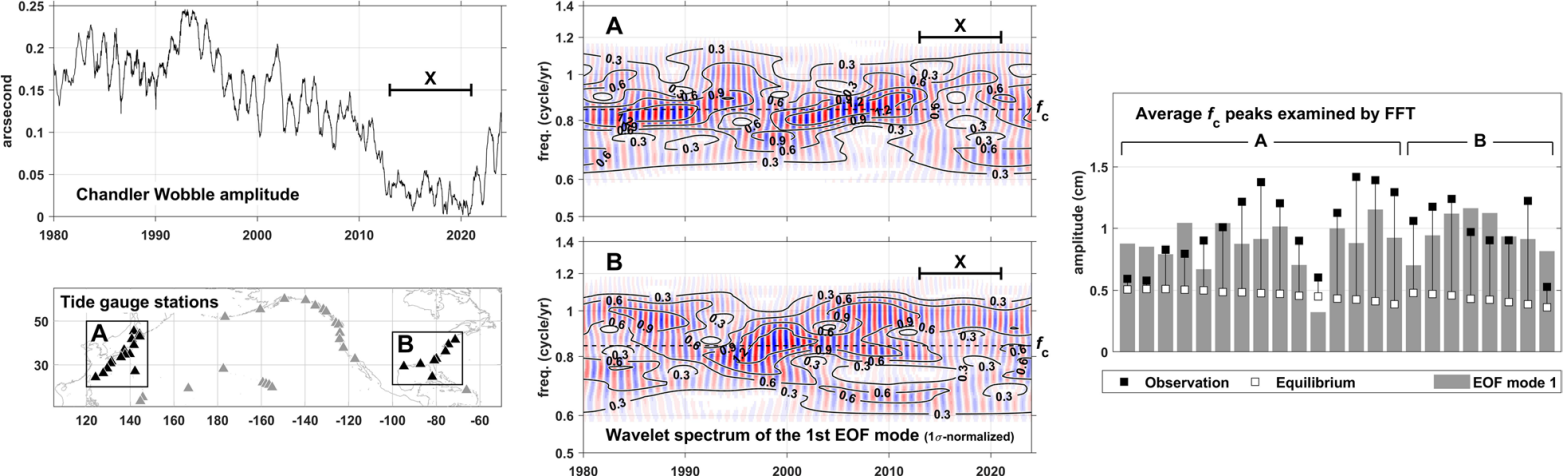

**Supplementary Information:**

The online version contains supplementary material available at 10.1186/s40623-026-02461-4.

## Introduction

Changes in centrifugal potential at Earth’s surface due to the periodic motion of Earth’s rotational pole cause sea-level changes, referred to as the ocean pole tide. The two dominant polar motion components, the annual wobble and the Chandler Wobble (CW), force pole tides at periods of 1 year and about 14 months, respectively. While the annual pole tide is obscured by many other causes of annual sea-level change, the distinct 14-month CW component has been the subject of many previous studies (Desai 2002; Miller and Wunsch 1973; Munk and MacDonald 1960; Wahr et al. 2015; Plag 1997).

Response of the oceans due to polar motion is expected to conform to equilibrium pole tide theory (Munk and MacDonald 1960). In recent studies, this theory is understood to be displacement of the ocean surface conforming to a deformed equipotential, considering combined effects of (1) changes in centrifugal potential due to polar motion, (2) conservation of ocean mass, and (3) self-attraction and loading of ocean mass redistribution (Desai 2002). Polar motion with a typical amplitude of 100 milliarcseconds (mas) produces an equilibrium pole tide with a maximum amplitude of approximately 4–5 mm at 45° latitudes. Previous studies using satellite altimetry (Desai 2002) and global tide gauge measurements (Trupin and Wahr 1990) found that the observed pole tide agrees relatively well with equilibrium theory on a global scale. However, it is also known that regional pole tide variations often differ significantly from equilibrium. For example, a 14-month tidal variation in the North and Baltic Seas is several times larger than equilibrium, likely due to local effects unrelated to polar motion, including meteorological forcing, local resonance, friction, and others (Miller and Wunsch 1973; O’Connor et al. 2000; Trupin and Wahr 1990; Xie and Dickman 1996). Local effects unrelated to polar motion may also be the cause of variability in the dominant frequency which ranges from ~ 0.7 to ~ 0.9 cycles per year (cpy) (Medvedev et al. 2017; Naito 1977; Plag 1997).

The CW amplitude diminished considerably after ~ 2015 (Yamaguchi and Furuya 2024). A similar amplitude reduction event was observed in the period 1925–1940 (Fedorov and Yatskiv 1965; Guinot 1972; Vondrak and Ron 2005). The recent CW amplitude reduction is expected to diminish the 14-month pole tide, raising the question of whether this effect can be detected in observations such as satellite altimetry or tide gauges. Because satellite altimetry products are distributed with pole tide corrections applied, following Desai (2002), we instead examine in this study sea-level change recorded at tide gauges.

Isolating pole tide signals from individual tide gauge records is challenging because amplitudes are relatively small compared to other sources of sea-level variability. Moreover, as noted earlier, the pole tide response at individual stations can deviate from equilibrium in both frequency and amplitude due to various regional effects. Trupin and Wahr (1990) considered a stacking of tide gauge measurements, which effectively amplifies the pole tide signal and yields a representative mean response over a study region. This approach provides a spatially averaged signal, but does not reveal behavior of individual stations. In this study, we instead applied Empirical Orthogonal Function (EOF) analysis, which not only produces a leading mode similar to the stacked signal, but also provides spatial contributions that reveal regional coherence as will be shown in Sect. 3.

The pole tide should appear as a relatively weak signal with strong spatial coherence across a regional array of tide gauges. As explained in Sect. 2, we applied a band-pass filter to isolate low-frequency sea-level variations near the CW frequency (${f}_{\mathrm{c}}$, approximately 0.8435 cpy), prior to EOF analysis. When equilibrium response is indeed significant in a studied region, the regional pole tide signal is expected also to show a decrease in amplitude during the period 2015–2020. Results in Sect. 3 demonstrate that the leading EOF modes effectively separate correlated low-frequency sea-level variations near ${f}_{\mathrm{c}}$. More importantly, pole tide signals obtained from some regions, such as Japan, show an amplitude reduction, likely associated with the recent CW amplitude reduction after ~ 2015.

## Data and methods

### Tide gauge measurements

We used daily data from more than 300 global tide gauge stations provided by the University of Hawaii Sea Level Center (UHSLC) (Caldwell et al. 2015). Time series from each station span different periods with varying data gaps. We selected stations with data available from 1980 to 2024, and excluded stations with gaps exceeding two consecutive years. Gaps shorter than two years were interpolated using the median value in a moving window. Selected stations are mostly located in Japan, North America, Europe, and the Pacific islands (Additional file 1: Fig. S1). To isolate signals near ${f}_{\mathrm{c}}$, we first removed annual variations from each station time series using least squares, and then applied a band-pass filter. Specifically, we constructed an eighth-order Butterworth filter with a passband of 0.65–1.05 cpy and used its magnitude response to weight the Discrete Fourier Transform (DFT) coefficients of each time series. The resulting band-limited tide gauge measurement array (hereafter denoted as $\Delta \mathrm{H}$) contains pole tide signals together with other low-frequency components in this passband.

### Polar motion and the equilibrium pole tide

**Fig. 1:**
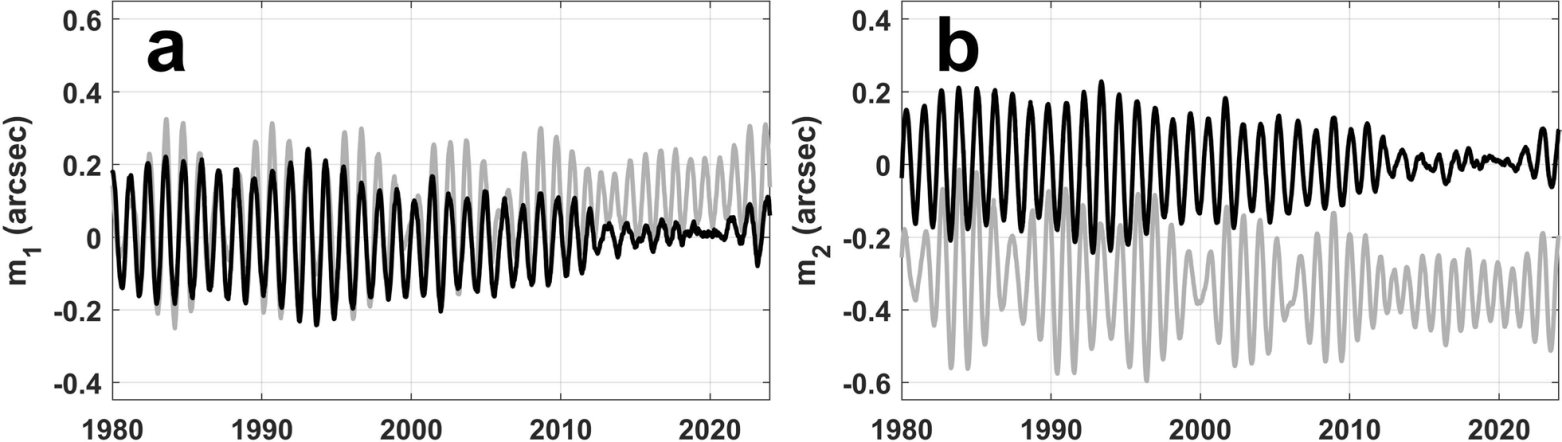
Polar motion for 1980–2024. **a** The *x*-component of observed polar motion (gray) and the CW variation (black). **b** Similar to **a**, but for the *y*-component

The Earth Orientation Parameters (EOP) C04 data from the International Earth Rotation and Reference Service (IERS) (Bizouard et al. 2019) provide daily polar motion values from 1962 to the present, consistent with the International Terrestrial Reference Frame (ITRF) 2020. The *x*- and *y*-components denoted as ${m}_{1}$ and ${m}_{2}$, positive toward 0° and 90° East longitude, respectively, and the changes from 1980.0 to 2024.0 are indicated as gray curves in Fig. 1. We obtained the CW variation by removing the linear trend, mean value, and annual and semi-annual variations (the semi-annual change is quite small) from ${m}_{1}$ and ${m}_{2}$ by least squares (black curves in Fig. 1a, b). The CW time series are dominated by a near 433-day periodicity, with significantly reduced amplitude after ~ 2015 (Yamaguchi and Furuya 2024).

The equilibrium pole tide (denoted as $\Delta \mathrm{E}$) at colatitude $\theta$ and east longitude $\phi$ considers a sea-level change conforming to an equipotential, resulting from polar motion:

1 $$\Delta \mathrm{E}\left(\theta , \phi \right)=-\frac{\left(1+{k}_{2}-{h}_{2}\right)}{\sqrt{15}}\frac{{\Omega }^{2}{a}^{2}}{g}\left({m}_{1}\left(t\right) {U}_{1}\left(\theta , \phi \right)+{m}_{2}\left(t\right) {U}_{2}\left(\theta , \phi \right)\right) O\left(\theta , \phi \right)$$

where ${k}_{2}$ and ${h}_{2}$ are degree-2 tidal Love numbers, $\Omega$ is the mean angular velocity of Earth’s rotation, and $a$ and $g$ are mean radius and gravitational acceleration of Earth, respectively. The solution is obtained over the oceans, whose domain is defined by the ocean function $O\left(\theta , \phi \right)$ (Munk and MacDonald 1960). ${U}_{1}\left(\theta ,\phi \right)$ and ${U}_{2}\left(\theta ,\phi \right)$ are the unit responses of sea-level change due to *x*- and *y*-directional polar motion, respectively, which include effects from centrifugal potential change, and ocean mass redistribution considering self-gravitation, loading, and mass conservation (Desai 2002; Adhikari et al. 2016). We computed $\Delta \mathrm{E}$ from the CW time series (black curves in Fig. 1a, b) at all tide gauge station locations. $\Delta \mathrm{E}$ is well approximated by degree-2 and order-1 spherical harmonics, although there are small differences associated with self-attraction and loading. As a result, the equilibrium pole tide $\Delta \mathrm{E}$ at each station is nearly linearly proportional to the CW time series in Fig. 1, with amplitude and phase varying among stations. However, pole tide signals observed at tide gauges likely exhibit more complex behavior than an equilibrium response, and likely do not scale linearly as well. Therefore, we examine whether regional low-frequency sea-level variability contributes to reduced amplitudes at frequencies near ${f}_{\mathrm{c}}$.

### EOF analysis

At individual stations, the pole tide component contained in $\Delta \mathrm{H}$ can exhibit different amplitudes and phases. When substantial phase differences exist across stations, ordinary EOF analysis cannot reliably extract a coherent pole tide pattern; in such cases, alternative approaches, such as complex EOF [e.g., Yu et al. (2018)], would be needed to properly account for propagating signals. In our case, tide gauge stations are distributed sparsely, making it difficult to resolve systematic propagation patterns directly from the array. Within sufficiently small regions, however, pole tide phases are expected to be approximately the same, so these regional signals behave effectively as standing oscillations. Under this condition, ordinary EOF analysis can extract a stable regional mode that should include the expected pole tide variability.

Thus, we grouped tide gauge stations into regional subsets, and constructed a matrix $\mathrm{D}$, where the *s*-th column represents $\Delta \mathrm{H}$ time series from the *s*-th tide gauge station. The matrix $\mathrm{D}$ was then decomposed into multiple EOF modes such as (Eom et al. 2017; Oh et al. 2025; Navarra and Simoncini 2010)

2 $$\mathrm{D}\left(t, s\right)=\sum_{m}{\mathrm{T}}_{m}\left(t\right) {\mathrm{S}}_{m}^{*}\left(s\right)$$

where $t$ and $s$ represent time and station indices, respectively, and asterisk (*) denote matrix transpose. ${\mathrm{S}}_{m}$ and ${\mathrm{T}}_{m}$ are column vectors representing respectively the spatial pattern (loading vector) and temporal variation (principal component) of the *m*-th EOF mode.

Because EOF modes tend to be biased by stations with a more variable sea level, normalization of $\mathrm{D}$ is widely used (Navarra and Simoncini 2010) so that matrix $\mathrm{D}$ contains normalized column vectors

3 $$\mathrm{D}\left(t,s\right)=\left[\begin{array}{cc}\begin{array}{cc}\frac{1}{\sigma \left[\Delta {\mathrm{H}}_{1}\right]}\Delta {\mathrm{H}}_{1}\left(t\right),& \frac{1}{\sigma \left[\Delta {\mathrm{H}}_{2}\right]}\Delta {\mathrm{H}}_{2}\left(t\right),\end{array}& \begin{array}{cc}\dots ,& \frac{1}{\sigma \left[\Delta {\mathrm{H}}_{\mathrm{s}}\right]}\Delta {\mathrm{H}}_{\mathrm{s}}\left(t\right)\end{array}\end{array}\right]$$

where $\Delta {\mathrm{H}}_{\mathrm{s}}$ is the un-normalized column vector from the *s*-th tide gauge station, and $\sigma \left[\right]$ indicates the standard deviation of that station.

In the EOF results presented below, we defined ${\mathrm{T}}_{m}$ as a dimensionless temporal component with unit standard deviation and scaled ${\mathrm{S}}_{m}$ accordingly. Because of the normalization in Eq. 3, the product of ${\mathrm{T}}_{m}{\mathrm{S}}_{m}^{*}$ represents the 1σ-normalized variation of the *m*-th mode. In the figures, we present the fully scaled ${\mathrm{S}}_{m}$ (e.g., $\upsigma \left[\Delta {\mathrm{H}}_{s}\right]\cdot {\mathrm{S}}_{m}$), so that the *m*-th mode reconstruction has the correct physical units. Pole tide signals were identified based on the leading modes that exhibit (1) significant spectral power in ${\mathrm{T}}_{m}$ near ${f}_{\mathrm{c}}$, and (2) spatial patterns in ${\mathrm{S}}_{m}$ coherent across the study area.

Alternatively, previous studies considered a weighted sum of station time series to enhance relatively weak pole tide signals (e.g., Trupin and Wahr (1990)). For comparison, we constructed a simplified stacked time series based on the band-pass-filtered $\Delta \mathrm{H}$, normalized by their standard deviation:

4 $$\mathrm{K}\left(t\right)=\sum_{s}\frac{1}{\sigma \left[\Delta {\mathrm{H}}_{\mathrm{s}}\right]}\Delta {\mathrm{H}}_{s}\left(t\right)$$

This stacked time series $\mathrm{K}\left(t\right)$ is further scaled to have unit standard deviation for consistency with the EOF temporal modes ${\mathrm{T}}_{m}\left(t\right)$.

5 $$\mathrm{K}^{\prime}\left(t\right)=\frac{\mathrm{K}}{\sigma \left[\mathrm{K}\right]}$$

Since ${\mathrm{T}}_{m}$ (and $\mathrm{K}^{\prime}$) capture all low-frequency variability retained in the passband, we applied the continuous wavelet transform to investigate the frequency-time variations of these signals. We employed analytic wavelet functions, which yield complex-valued wavelet spectra. In the figures below, we display the real part of spectrum, which reveals the typical phase structure through alternating positive and negative bands (Chao et al. 2014; Chao and Naito 1995). To aid interpretation of amplitude variations, we additionally overlay contour lines derived from the magnitudes of the complex coefficients.

## Results

### Comparison of stacking method and EOF analysis

**Fig. 2:**
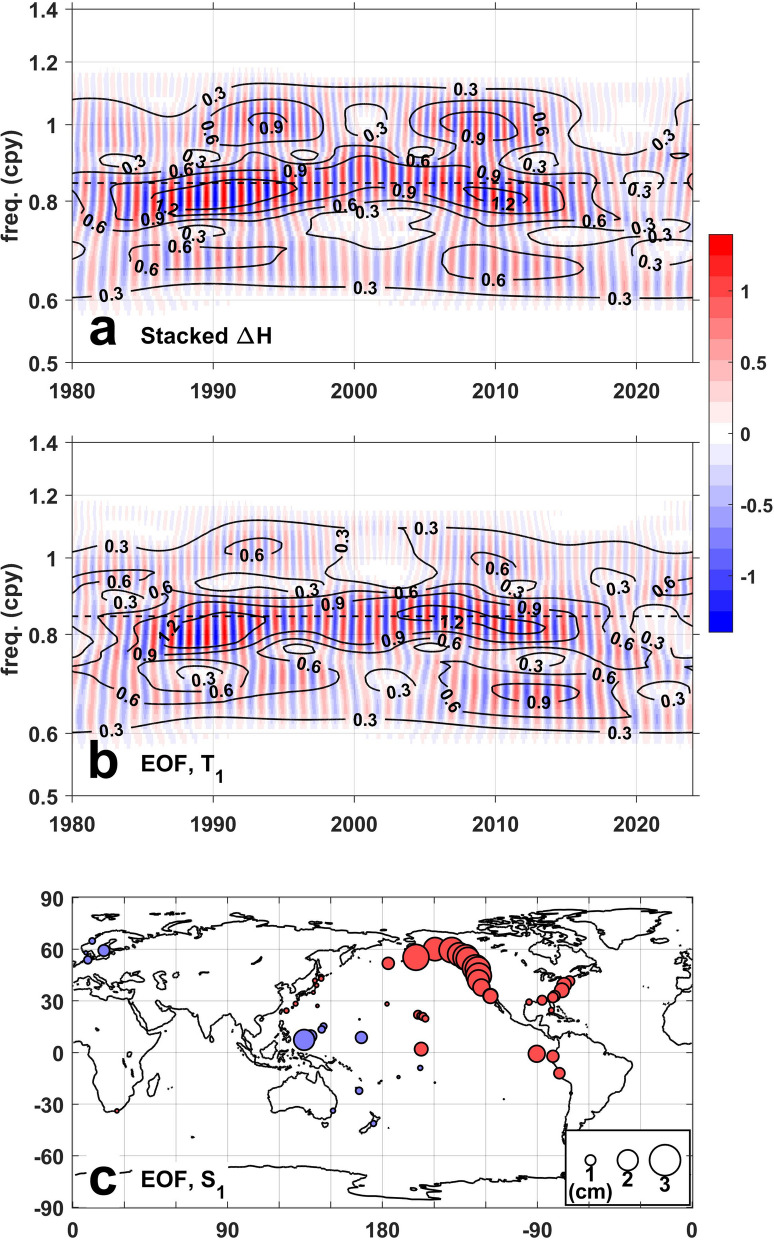
Comparison of the stacking method and EOF analysis using 71 global tide gauge stations (see Figure S1 for station locations). **a** Wavelet spectrum of globally stacked tide gauge measurement $\mathrm{K}^{\prime}$. **b** Wavelet spectrum of temporal pattern of EOF mode 1 (i.e., ${\mathrm{T}}_{1}$). In both **a**, **b** the blue and red stripes indicate the troughs and crests of the real part of the wavelet spectrum, respectively. The overlaid contours indicate the magnitude of the wavelet coefficients, and the dashed horizontal line indicates the CW frequency ${f}_{\mathrm{c}}$, ~ 0.8435 cpy. **c** Spatial pattern of EOF mode 1 (${\mathrm{S}}_{1}$). The radius of the circle is proportional to the station amplitude, with negative and positive signs indicated by blue and red, respectively. All tide gauge measurements were band pass-filtered and normalized prior to analysis (see Sect. 2.1)

We examined the stacked time series (i.e., $\mathrm{K}^{\prime}$) and leading EOF modes (${\mathrm{T}}_{1}$ and ${\mathrm{S}}_{1}$) using 71 globally distributed tide gauge stations, recognizing that pole tide signals with opposite phases at different locations may cancel during summation and thereby hinder the extraction of a globally coherent mode. The wavelet spectrum of $\mathrm{K}^{\prime}$ (Fig. 2a) shows a relatively stable 14-month pole tide signal near ${f}_{\mathrm{c}}$ (indicated by the dashed horizontal line). A similar temporal pattern is extracted by EOF mode 1, which accounts for about 22% of the total variance (Fig. 2b), indicating that both approaches have effectively captured the same dominant variability. However, the spatial pattern of EOF mode 1 (Fig. 2c) reveals that this dominant variability arises primarily from stations along the North American coast, whereas stations in Japan, Europe, and other regions contribute very little to the mode. This suggests that, although both stacking and EOF mode 1 yield comparable temporal behavior, the resulting signal tends to reflect regions with dense station coverage and minimal phase cancelation, rather than representing a truly global response.

Together, these results highlight the difficulty of extracting a globally coherent pole tide signal from tide gauge measurements and motivate a regional analysis. For EOF (or stacking) analysis to capture an effective mean pole tide pattern, the analysis domain should not be excessively large while maintaining sufficient station density. An overly large region can be subject to phase cancelation of the pole tide signal, as shown in Fig. 2, whereas sparse station coverage weakens the extracted common variability. We therefore focused on mid-latitude regions, where the equilibrium amplitude is expected to be large and data coverage is adequate. We considered four regions (Japan, the west and east coasts of North America, and Europe), each with good station density within relatively compact mid-latitude domains. In contrast, stations at Pacific islands were excluded because of their sparse distribution over a vast area, together with the smaller equilibrium pole tide amplitude expected at low latitudes. In practice, Pacific island $\Delta \mathrm{H}$ time series are dominated by other low-frequency signals that vary substantially among stations (Additional file 1: Fig. S2), and no coherent pole tide pattern was found in the leading EOF modes.

### Regional EOF analysis

**Fig. 3:**
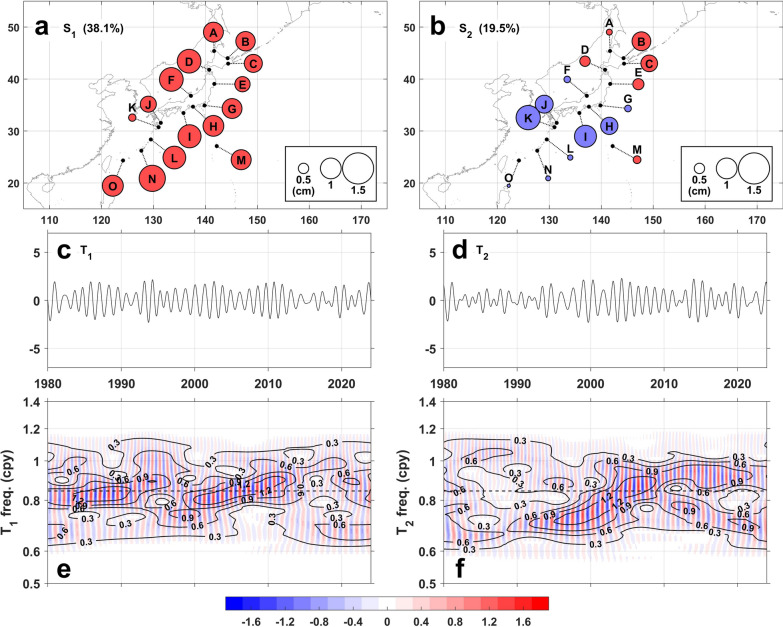
EOF modes 1 and 2 from 15 tide gauge stations A-O in Japan. **a**, **b** Spatial patterns ${\mathrm{S}}_{1}$ and ${\mathrm{S}}_{2}$, respectively. The black dots represent the tide gauge locations, connected with the colored circles visualizing the spatial patterns of the modes. The number in parenthesis is the explained variance of the mode. **c**, **d** Dimensionless temporal patterns ${\mathrm{T}}_{1}$ and ${\mathrm{T}}_{2}$, respectively. **e**, **f** Frequency–time spectra of ${\mathrm{T}}_{1}$ and ${\mathrm{T}}_{2}$ obtained by the wavelet transform

We first examined 15 stations in Japan. Figure 3 displays the first and second EOF modes. EOF mode 1 shows high spatial coherence across the entire tide gauge array, accounting for about 38% of total variance of the band-limited sea-level variations (Fig. 3a). The corresponding dimensionless temporal pattern ${\mathrm{T}}_{1}$, shown in Fig. 3c, was further analyzed using a wavelet transform (Fig. 3e). The resulting frequency-time spectrum reveals relatively strong power near ${f}_{\mathrm{c}}$. Note that the first and last ~ 5 years are possibly affected by wavelet edge effects. Nevertheless, the frequency-time spectrum of ${\mathrm{T}}_{1}$ exhibits a clear amplitude decrease during 2015–2020, which coincides with the CW amplitude reduction observed in polar motion (Yamaguchi and Furuya 2024).

EOF mode 2 (Fig. 3b) also includes power near ${f}_{\mathrm{c}}$, but the spatial pattern shows a phase difference between northern and southern parts of the array. This dipolar structure is unlikely to be related to the pole tide and likely reflects other effects. As shown in Fig. 3f, temporal variability of ${\mathrm{T}}_{2}$ is dominated by signals with frequencies above ~ 0.9 cpy (2005–2020) and below ~ 0.7 cpy (1990–2005). This is also unlikely to be associated with the CW because variations in the 0.8–0.9 cpy band are observed in the North and Baltic Seas, with significant pole tide modulation (e.g., Medvedev et al. (2017)). We also investigated higher modes (Additional file 1: Figs. S3 and S4). Mode 3 is associated with the southwestern islands of Japan (Fig. S3). Modes 4 and higher account for a small fraction of the total variance, and probably do not have a significant physical explanation as indicated by similar small explained variance magnitudes (Figure S4). In summary, the Japanese tide gauge array contains pole tide signals well correlated with the CW amplitude reduction event, as demonstrated by the frequency-time spectrum of EOF mode 1 (Fig. 3e). Note again that this pattern was not clearly recognized via the global stacking (Fig. 2a).

**Fig. 4:**
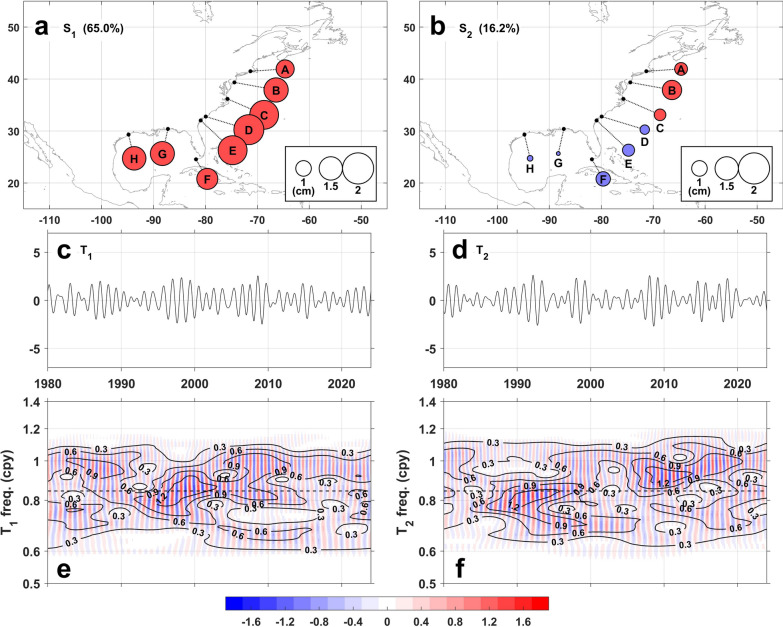
Similar to Fig. 3, but from 8 tide gauge stations along the east coast of North America

Among the selected tide gauge stations, 19 are located along the Atlantic and Pacific coasts of North America. EOF results using the 8 Atlantic stations are presented in Fig. 4. Mode 1 accounts for 65% of the low-frequency sea-level variance in this region, indicating that it represents the dominant pattern within the 0.65–1.05 cpy band (see also Figures S5 and S6 for the higher modes). The first EOF mode shows strong spatial coherence along the coastline (Fig. 4a). Although the signal is less pronounced than in the Japanese array, the temporal component ${\mathrm{T}}_{1}$ from this region still shows an amplitude decrease after ~ 2015 near ${f}_{\mathrm{c}}$ (Fig. 4e). Although an amplitude reduction is observed during the early period (1980–1985), this is likely contaminated by an artifact of wavelet edge effects.

**Fig. 5:**
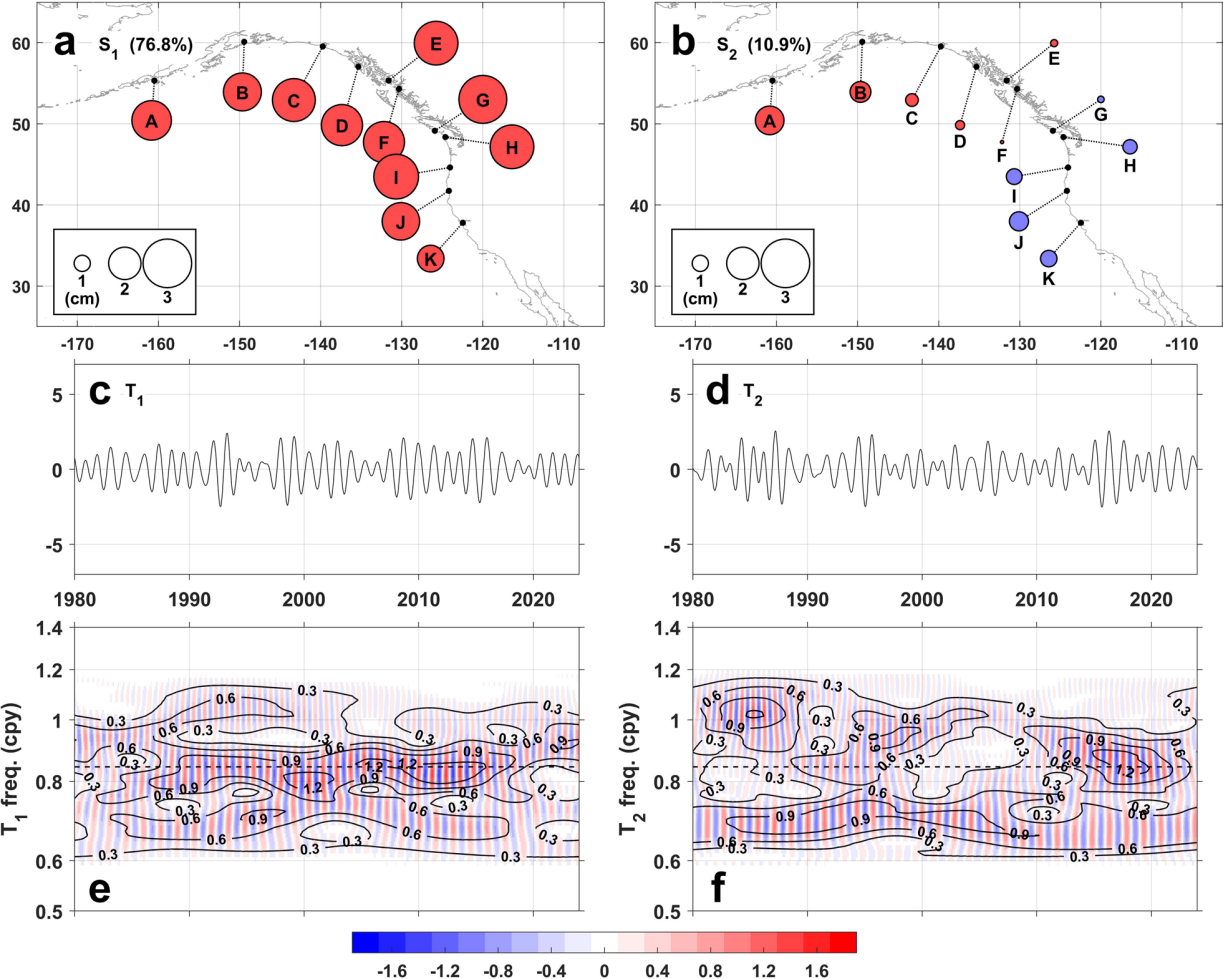
Similar to Fig. 3, but from 11 tide gauge stations along the west coast of North America

The tide gauge array along the Pacific coast of North America also exhibits strong spatial coherence within the region (Fig. 5). The first EOF mode shows a temporal pattern containing signals with significant amplitudes and stable frequency centered near ${f}_{\mathrm{c}}$ (Fig. 5e). Mode 1 explains about 77% of the variance in this region, with much less associated with other modes (see also Figs. S7 and S8). However, mode 1 does not show strong evidence of a pole tide reduction after 2015. Some reduction in amplitude appears around 2020, but as noted above, this may reflect a delayed pole tide response or be an artifact of wavelet edge effects. Additional observational data will be necessary to clarify this. The data used in this study indicate that tide in this region does not appear related to the CW variation.

**Fig. 6:**
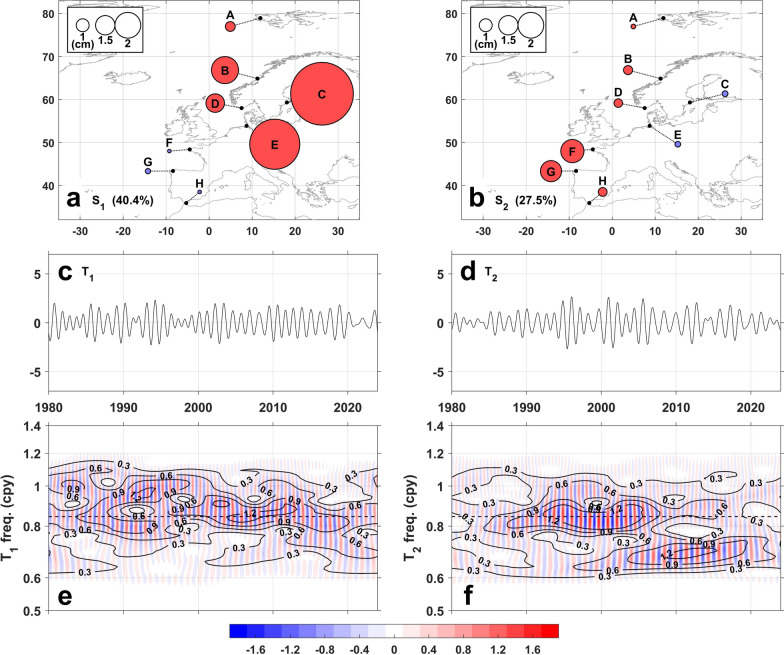
Similar to Fig. 3, but from 8 tide gauge stations in Europe

Lastly, we examined 8 stations in Europe, with EOF modes 1 and 2 presented in Fig. 6. EOF analysis did not capture a mode common to all 8 stations. Mode 1 is dominated by variations in the North and Baltic Seas (stations B-E in Fig. 6a), whereas mode 2 is stronger in southwestern areas (stations F and G in Fig. 6b). Apparently, the North and Baltic Seas have distinctive low-frequency sea-level variations near ${f}_{\mathrm{c}}$, as found in earlier studies (Medvedev et al. 2017; Miller and Wunsch 1973; Naito 1977; O’Connor et al. 2000; Xie and Dickman 1996). In the frequency–time spectrum of mode 1 (Fig. 6e), the signal near ${f}_{\mathrm{c}}$ does not appear to decrease in amplitude after 2015. This indicates that the North and Baltic Sea signals are probably not associated with the CW. On the other hand, mode 2 (Fig. 6f) shows an amplitude decrease, but the timing, somewhat earlier than 2010, is unlikely to be related to the recent CW amplitude reduction event. Mode 3 captures signals at a couple of stations (station A and H), and higher modes account for only a small fraction of the total variance (Additional file 1: Figs. S9 and S10).

### Pole tide amplitude

EOF mode 1 for Japan and the east coast of North America separates pole tide signals diminishing in amplitude after ~ 2015. Although this mode may also include non-equilibrium variations near ${f}_{\mathrm{c}}$, it should provide an improved estimate of the pole tide pattern across these regions. We reconstructed band-limited sea-level time series at each station from EOF mode 1, such as $\sigma \left[\Delta {\mathrm{H}}_{\mathrm{s}}\right]\cdot {\mathrm{T}}_{1}{\mathrm{S}}_{1}^{*}$ at the *s*-th tide gauge station. Among the signals within the 0.65–1.05 cpy passband, we used the amplitude of the DFT value nearest ${f}_{\mathrm{c}}$ as an estimate of average pole tide amplitude since 1980 at each station.

**Fig. 7:**
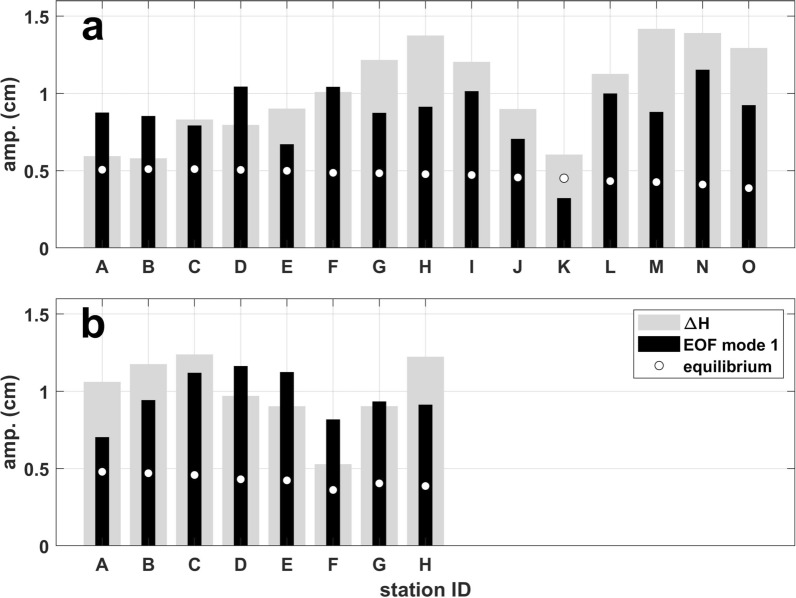
Pole tide amplitudes estimated at 15 tide gauge stations in Japan (**a**) and 8 stations along the east coast of North America (**b**). The locations of stations labeled A, B, C, etc. are shown in Figs. 3 and 4. The gray bars indicate the amplitude of ${f}_{\mathrm{c}}$ obtained from the DFT of ΔH, while the black bars show the ${f}_{\mathrm{c}}$ amplitude from the DFT of the time series reconstructed from EOF mode 1. The white dot represents the ${f}_{\mathrm{c}}$ amplitude based on the theoretical ΔE time series at each station (Eq. 1), which is normally smaller than 0.5 cm

Figure 7 summarizes these pole tide amplitude estimates from 15 stations in Japan (Fig. 7a), and 8 stations along the east coast of North America (Fig. 7b). Amplitudes from the DFT of the original tide gauge time series (i.e., $\Delta \mathrm{H}$, gray bars) vary considerably among stations, ranging from 0.5 to 1.5 cm, reflecting probable contamination by other signals. In contrast, estimates from DFT values using EOF mode 1 reconstruction (black bars) are mostly within 0.5–1 cm, suggesting an improved representation of the pole tide amplitude. These values are averages over the study period (1980–2024), and would include both equilibrium and non-equilibrium pole tide variations at each station. Further, these EOF-based estimates are generally larger than equilibrium values (white dots). Amplitudes exceeding the equilibrium may be affected by other non-equilibrium effects. On average, EOF-based pole tide amplitudes are approximately 1.8 times larger than the equilibrium. Previous studies found that regional amplitudes in the North and Baltic Seas can exceed 3 times the equilibrium value (Naito 1977; Plag 1997; Medvedev et al. 2017), whereas stations elsewhere have amplitudes close to equilibrium (Trupin and Wahr 1990). Considering these earlier results, our EOF-based estimates for both mid-latitude regions appear reasonable.

## Discussion and conclusions

Pole tide amplitudes are expected to decrease in response to the recent CW amplitude reduction event, and we investigated whether regional pole tide signals have changed accordingly. Extracting a global-scale pole tide pattern is challenging due to the propagating nature of pole tide signals, sparse tide gauge station density, and other forces that affect sea-level variations near ${f}_{\mathrm{c}}$. When a tide gauge array is examined over a region with sufficient station density and small phase differences, EOF analysis can effectively separate pole tide signals from other regional low-frequency variations.

We found that tide gauge arrays from Japan and the east coast of North America contain a 14-month pole tide signals whose amplitude decreases coincident with the CW amplitude reduction after 2015. In contrast, sea-level variability near ${f}_{\mathrm{c}}$ on the west coast of North America and in North and Baltic Seas do not appear directly related to the CW. Along the west coast of North America, EOF mode 1 includes a strong signal near ${f}_{\mathrm{c}}$, although its decreasing amplitude is not temporally consistent the recent CW reduction event. Sea level variability near ${f}_{\mathrm{c}}$ in North and Baltic Seas also shows no clear correspondence with the CW, consistent with many previous studies.

Although we examined sea-level variations near ${f}_{\mathrm{c}}$ in several regions using EOF signal separation and wavelet analysis of leading modes, precise quantification of the pole tide remains challenging. Observed changes ($\Delta \mathrm{H}$) likely contain a mixture of (1) equilibrium response that is almost linearly proportional to the CW amplitude, (2) non-equilibrium pole tide variability near ${f}_{\mathrm{c}}$, and (3) other unrelated low-frequency signals that coincidentally occur close to ${f}_{\mathrm{c}}$. To estimate the amplitude of the pole tide, we examined DFT amplitudes reconstructed from EOF mode 1, assuming that the spectral peak at ${f}_{\mathrm{c}}$ primarily reflects components (1) and (2). The peak amplitudes based on this EOF reconstruction generally fall in the range 0.5–1 cm, and are likely represent reasonable estimates of the mean pole tide amplitude at each station over the study period.

On average, these estimates are somewhat larger than the equilibrium prediction. Several mechanisms have been proposed to explain locally amplified pole-tide signals, particularly in the North and Baltic Seas. For example, Wunsch (1986) suggested that a free oscillation resonant with a traveling forcing function could enhance the response, while O’Connor et al. (2000) argued that quasi-periodic wind stress components contribute to the observed amplitude. Xie and Dickman (1996) also concluded that meteorological forcing is the primary source of sea-level variability near ${f}_{\mathrm{c}}$ in these regions.

In other regions, similar processes may cause significant deviations from an equilibrium response. Even in regions where the pole tide variability broadly follows the expected temporal evolution of the CW, such as Japan and the east coast of North America, the amplitudes do not approach zero during the recent CW minimum and remain systematically larger than the equilibrium prediction (Figs. 2 and 3). As shown in Fig. 7, the time-mean pole tide amplitudes for 1980–2024 exceed the equilibrium values even in these regions, indicating that the observed signals likely contain a mixture of equilibrium response and additional non-equilibrium variability.

Among the mechanisms proposed to explain such departures, atmospheric forcing represents a plausible contributor (Furuya et al. 1997, 1996; Aoyama and Naito 2001; Aoyama et al. 2003; Wang et al. 2025). In semi-enclosed basins, such as the North and Baltic Seas, where meteorological forcing has long been considered dominant, variability near ${f}_{\mathrm{c}}$ appears to be largely non-equilibrium in character. If atmospheric forcing contributes more broadly, its influence would be expected to vary regionally, depending on oceanographic setting and basin geometry. In this view, regions, such as Japan and the east coast of North America, may represent cases where the non-equilibrium component is present but comparatively weaker, so that the temporal evolution of the pole tide still exhibits the equilibrium-like behavior with enhanced amplitudes. Although we do not directly test this mechanism, our results are qualitatively consistent with a continuum of behavior ranging from predominantly equilibrium-like responses (e.g., Japan) to strongly non-equilibrium variability (e.g., North and Baltic Seas) across different regions.

Earlier studies (Hosoyama et al. 1976; Naito 1977) also noted phase differences between the western and eastern North Pacific although underlying causes remain unclear. We similarly observe a delay in the northeastern Pacific array (Fig. 5), but additional observations would be required to determine whether this reflects a genuine physical propagation or instead is an artifact due to the wavelet edge effect. The pronounced CW amplitude reduction after 2015 may therefore provide a useful temporal marker for evaluating possible phase delays and propagation characteristics in future studies.

The present analysis, based solely on tide gauge measurements, highlights several limitations inherent in analyzing signals from sparse, irregularly distributed station arrays. Pole tide variability includes propagating components whose phase variations cannot be fully represented by ordinary EOF analysis when station spacing is large. Satellite altimetry, in contrast, provides relatively spatially continuous sea-level measurements and may therefore offer a more suitable dataset for investigating propagation characteristics of the non-equilibrium pole tide (e.g., via complex EOF). Because theoretical equilibrium pole tides are already removed in standard altimetry products (e.g., Pujol et al. (2016); Legeais et al. (2018); Watson et al. (2015)), any remaining 14-month variability may reflect non-equilibrium signals, which may also propagate. A careful reconstruction of these residuals could thus provide valuable insights complementary to those obtained from tide gauge arrays. Exploring this possibility would be an important direction for future work.

## Supplementary Information

Additional file 1.

## Data Availability

The EOP data from IERS are distributed from http://www.iers.org/IERS/EN/DataProducts/EarthOrientationData/eop.html. The tide gauge data processed by the UHSLC is available at https://uhslc.soest.hawaii.edu/data/.

## Abbreviations

CW: Chandler wobble
DFT: Discrete fourier transform
EOF: Empirical orthogonal functions
$${f}_{\mathrm{c}}$$: CW frequency
IERS: International earth rotation and reference service
ITRF: International terrestrial reference frame
UHSLC: University of hawaii sea level center
cpy: Cycles per year
mas: Milli-arc-seconds
