# Supplementary material for: Preliminary tide gauge evidence for equilibrium and non-equilibrium pole tide variability after the 2015 Chandler Wobble amplitude reduction
Source: Earth Planets Space. 2026 May 19;78(1):139. doi: 10.1186/s40623-026-02461-4 (PMC13350126; doi:10.1186/s40623-026-02461-4)
Supplement: Supplementary file 1 — Additional file 1. [file 40623_2026_2461_MOESM1_ESM.docx]

**Preliminary tide gauge evidence for pole tide reduction after 2015 associated with diminished Chandler Wobble amplitude**

Taehwan Jeon

Center for Educational Research, Seoul National University, Seoul, 08826, Republic of Korea, naiad123@snu.ac.kr

Ki-Weon Seo

Department of Earth Science Education, Seoul National University, Seoul, 08826, Republic of Korea, seokiweon@snu.ac.kr

Kookhyoun Youm

Division of Glacier and Earth Sciences, Korea Polar Research Institute, Incheon, 21990, Republic of Korea, khyoum@kopri.re.kr

Jooyoung Eom

Department of Earth Science Education, Kyungpook National University, Daegu, 41566, Republic of Korea, eomjy@knu.ac.kr

Jianli Chen

State Key Laboratory of Climate Resilience for Coastal Cities, Department of Land Surveying and Geo-Informatics, The Hong Kong Polytechnic University, Hong Kong, China / Research Institute for Land and Space, The Hong Kong Polytechnic University, Hong Kong, China / The Hong Kong Polytechnic University Shenzhen Research Institute, Shenzhen, China, jianli.chen@polyu.edu.hk

Clark R. Wilson

Center for Space Research, University of Texas at Austin, Austin, TX 78759, USA / Department of Earth and Planetary Sciences, Jackson School of Geosciences, University of Texas at Austin, Austin, TX 78712, USA, crwilson@jsg.utexas.edu

**Corresponding author:** Taehwan Jeon


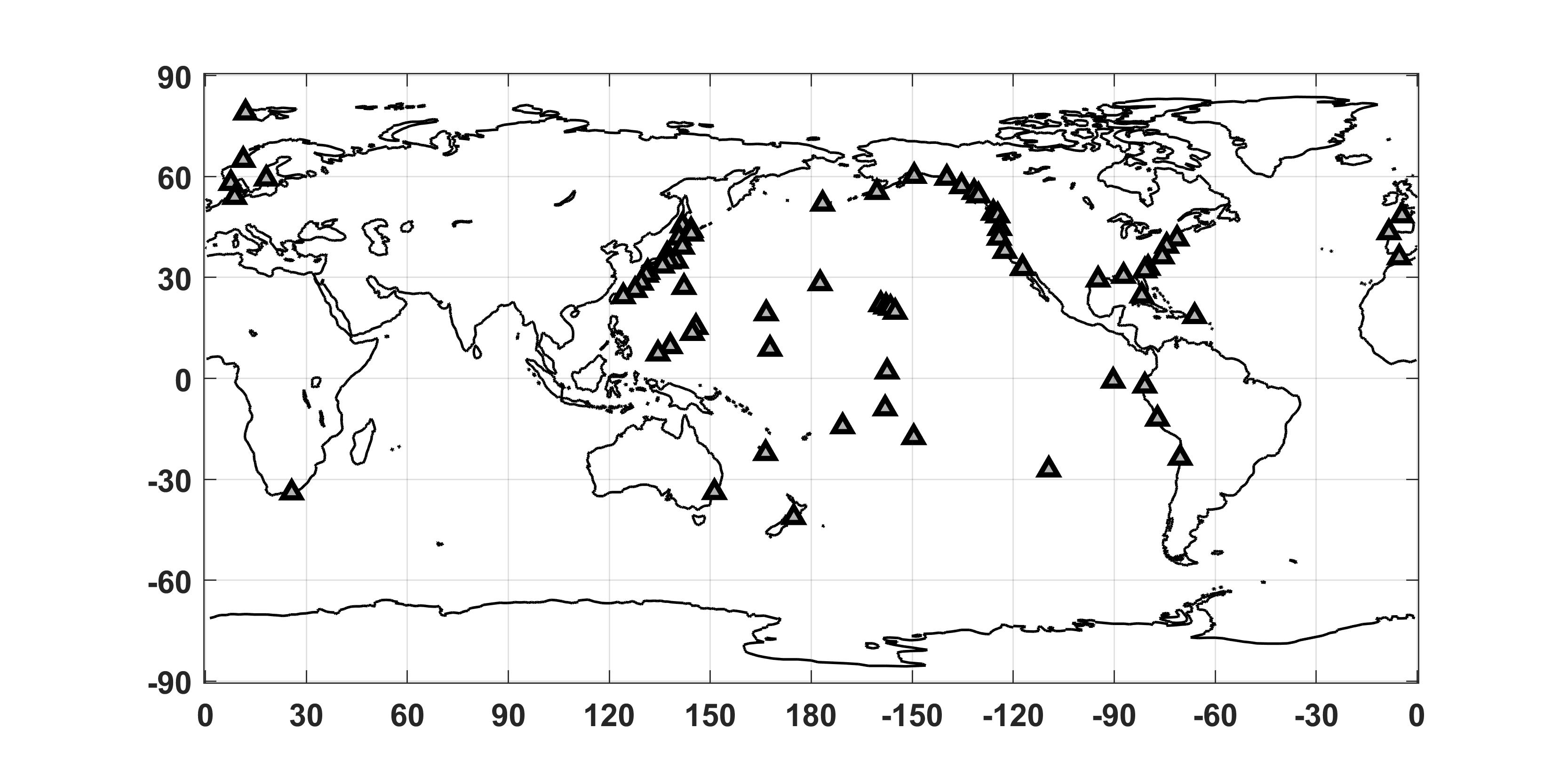


**Figure S1.** Locations of the 71 tide gauge stations used in this study (triangles). All stations provide sea level observations since 1980, with no data gaps longer than two consecutive years.


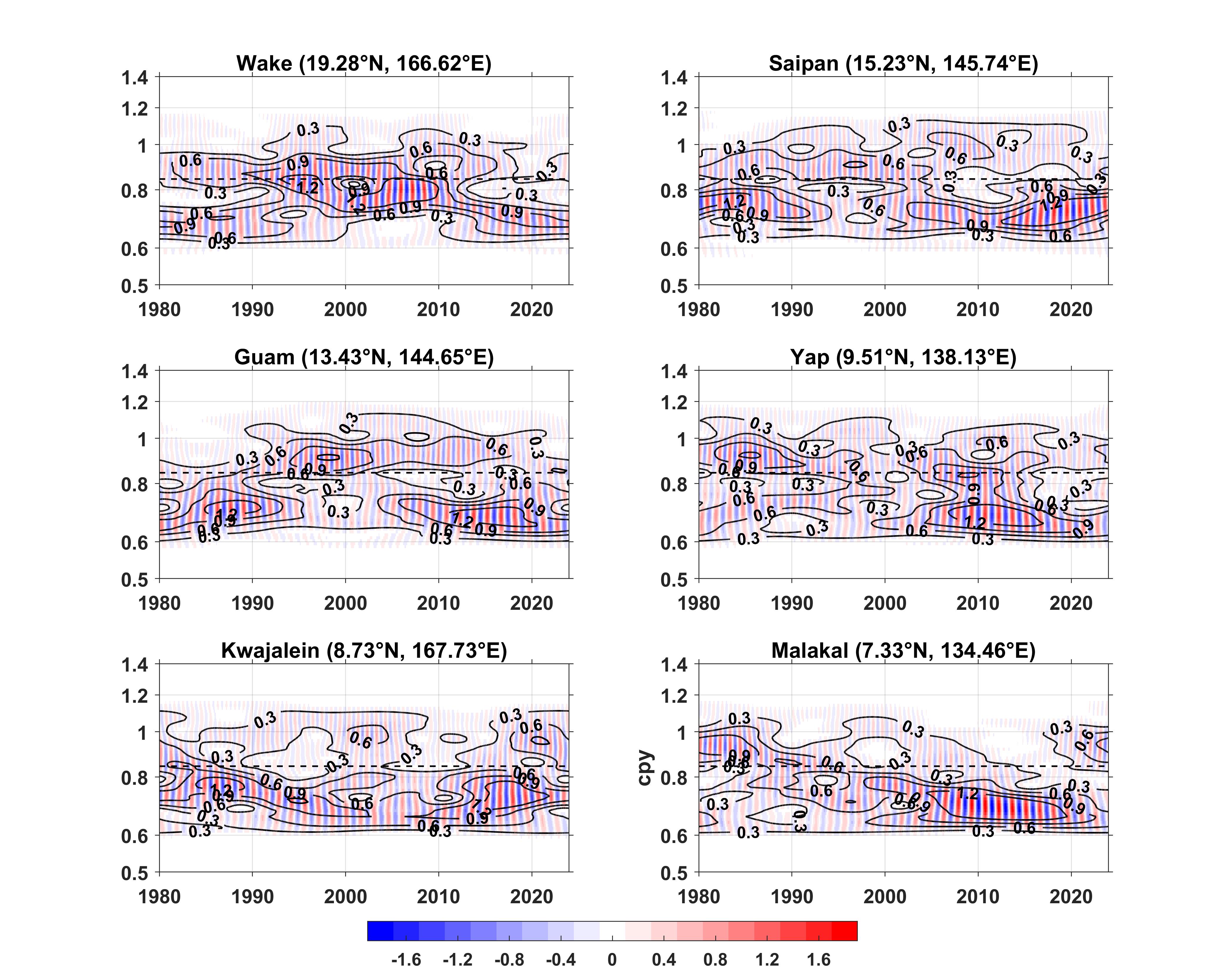


**Figure S2.** Wavelet transform frequency-time spectra of $\Delta H$ from tide gauge stations (1σ normalized) in the western Pacific region.

**
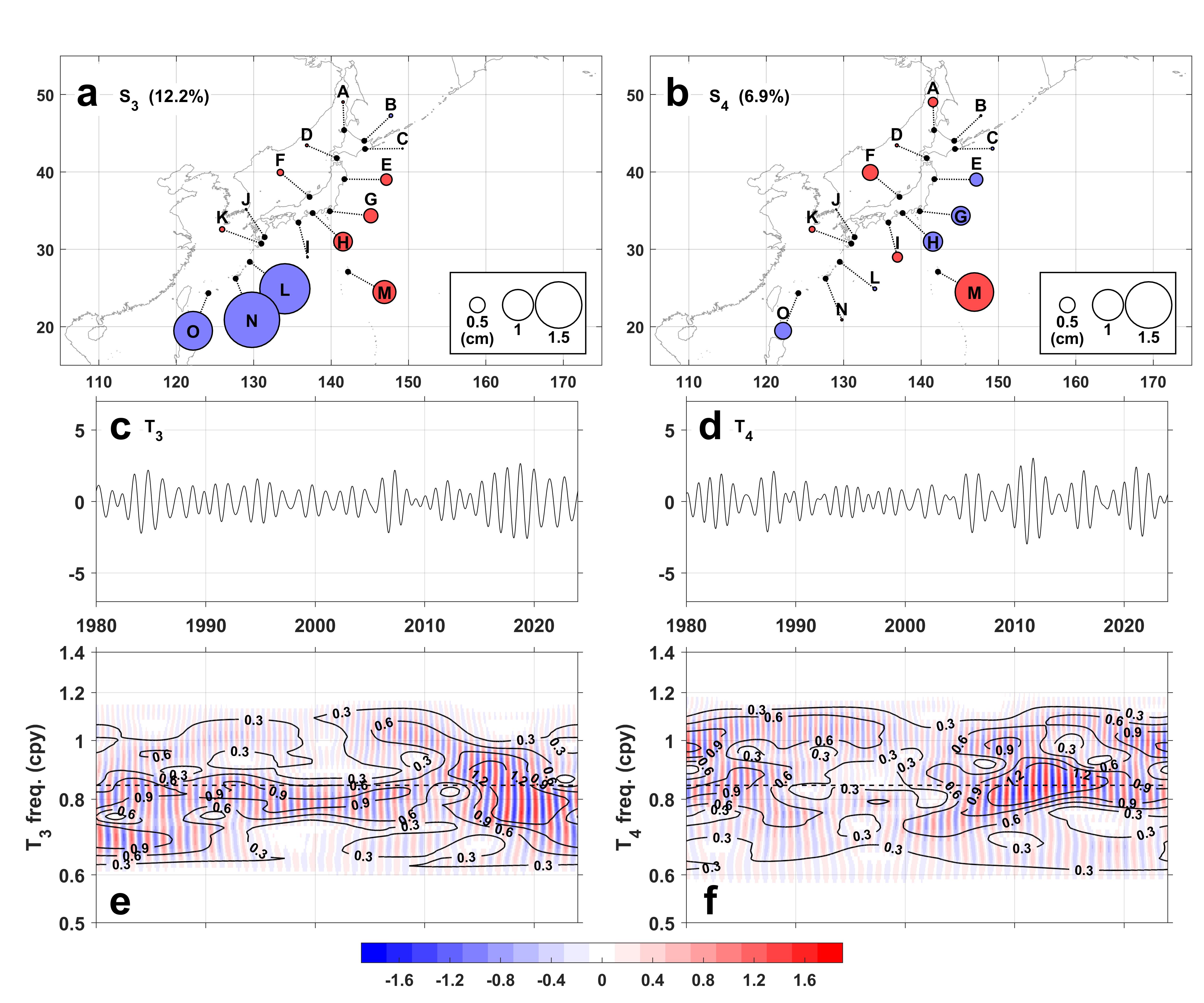
**

**Figure S3.** Similar to Fig. 3 in the main text, but for EOF modes 3 and 4.


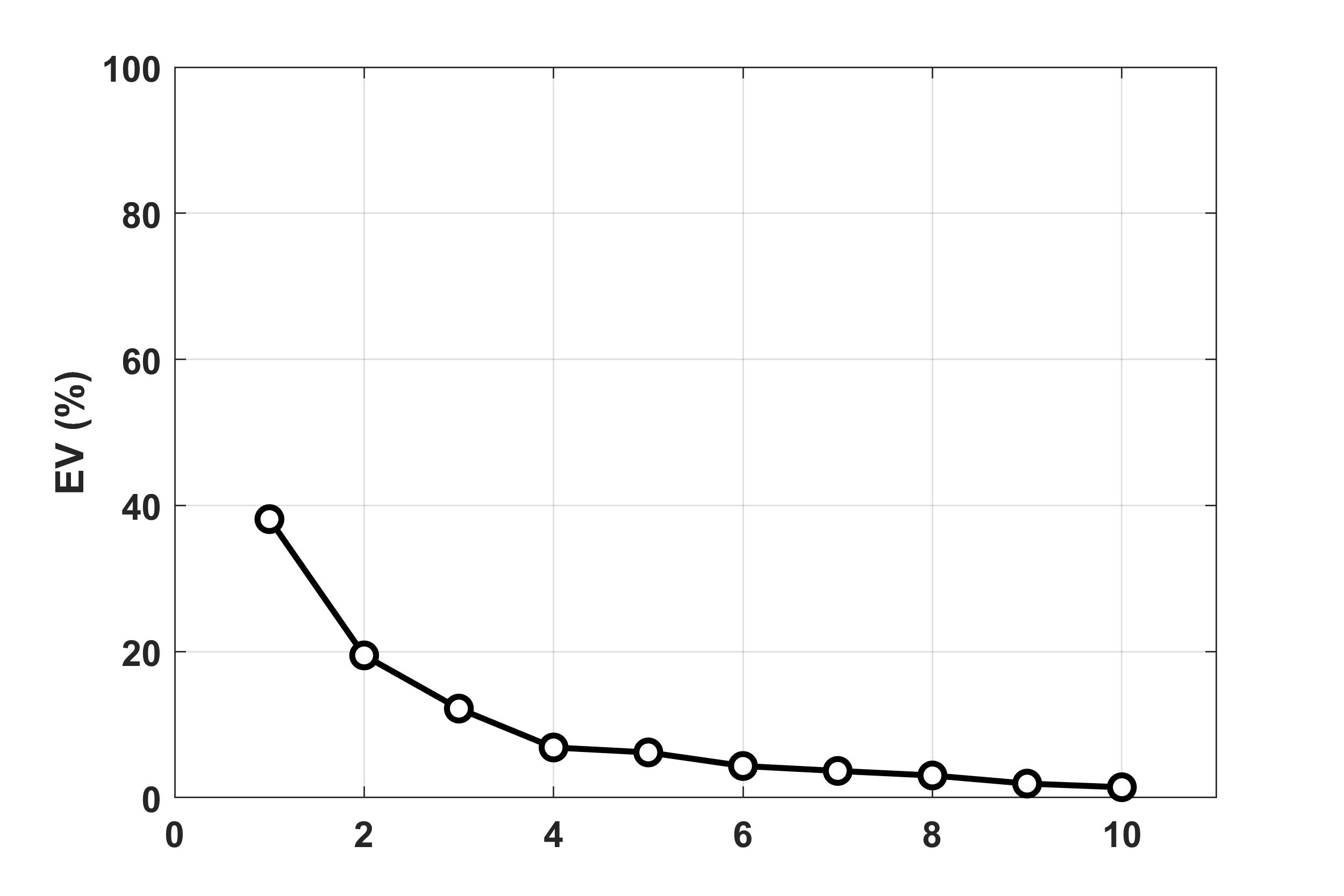


**Figure S4.** Explained variances (EV) of the EOF modes up to 10 derived from 15 stations in Japan.


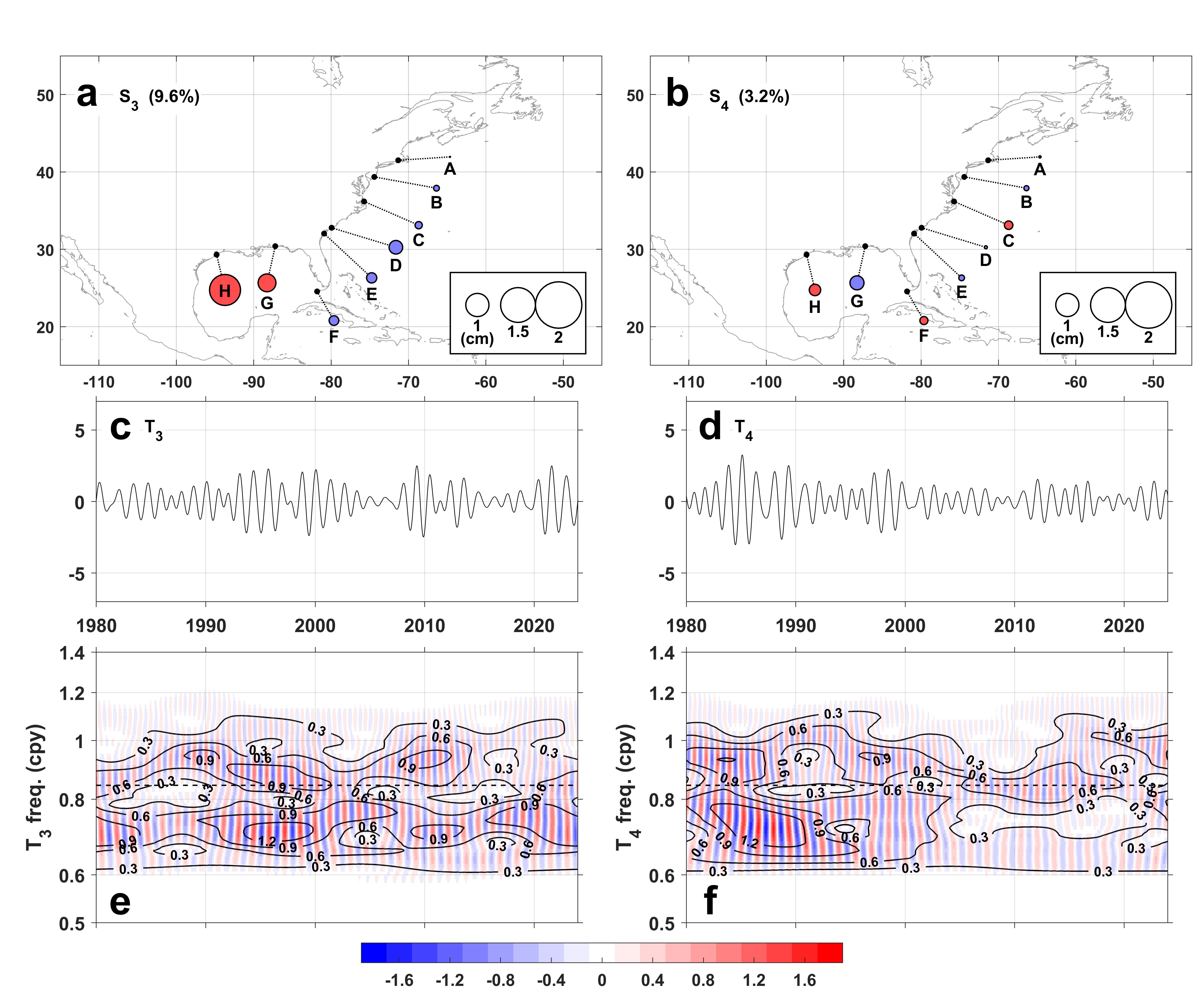


**Figure S5.** Similar to Fig. 4 in the main text, but for EOF modes 3 and 4.


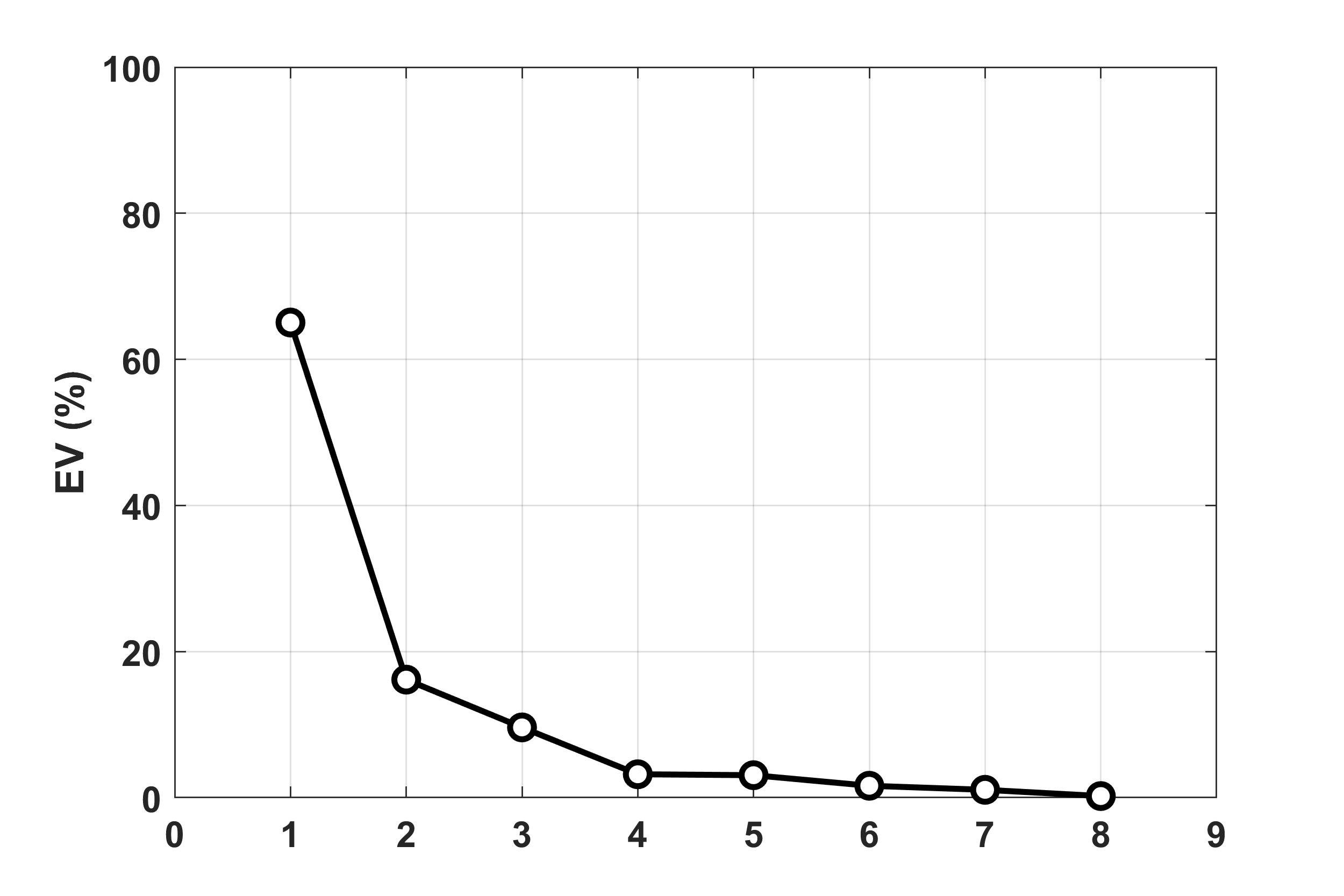


**Figure S6.** Explained variances (EV) of the EOF modes up to 8 derived from 8 stations along the eastern coastline of North America.


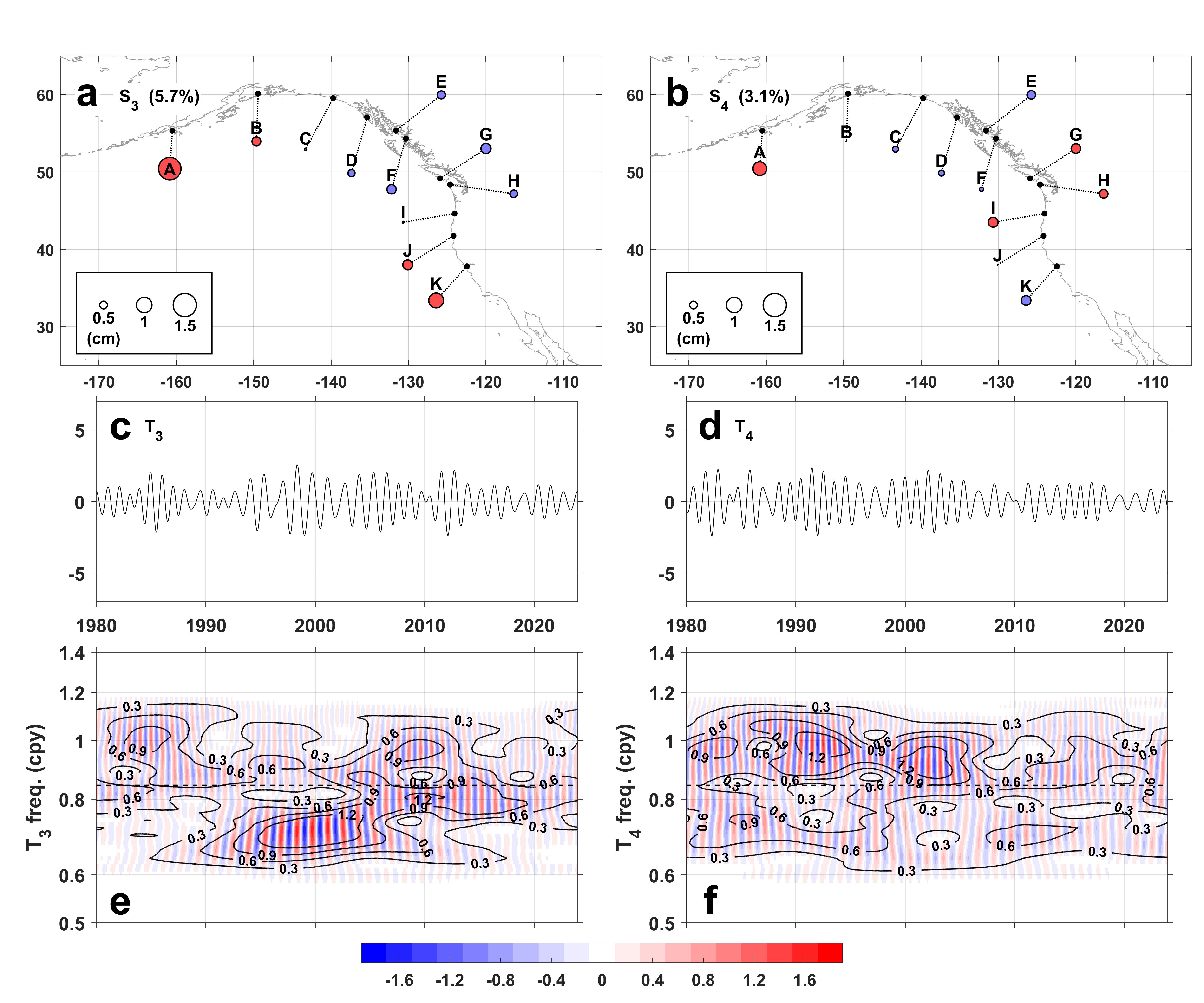


**Figure S7.** Similar to Fig. 5 in the main text, but for EOF modes 3 and 4.


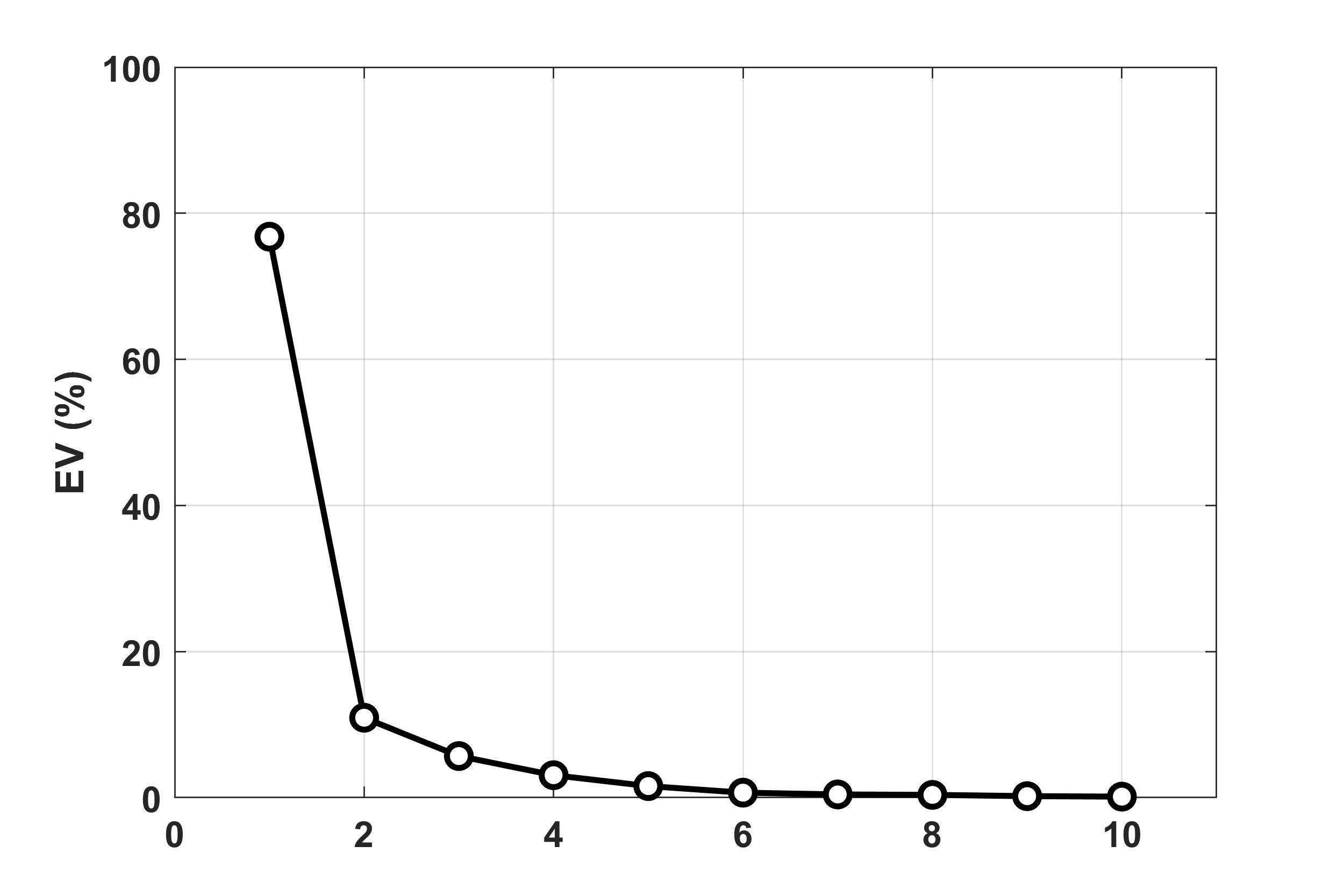


**Figure S8.** Explained variances (EV) of the EOF modes up to 10 derived from 11 stations along the western coastline of North America.


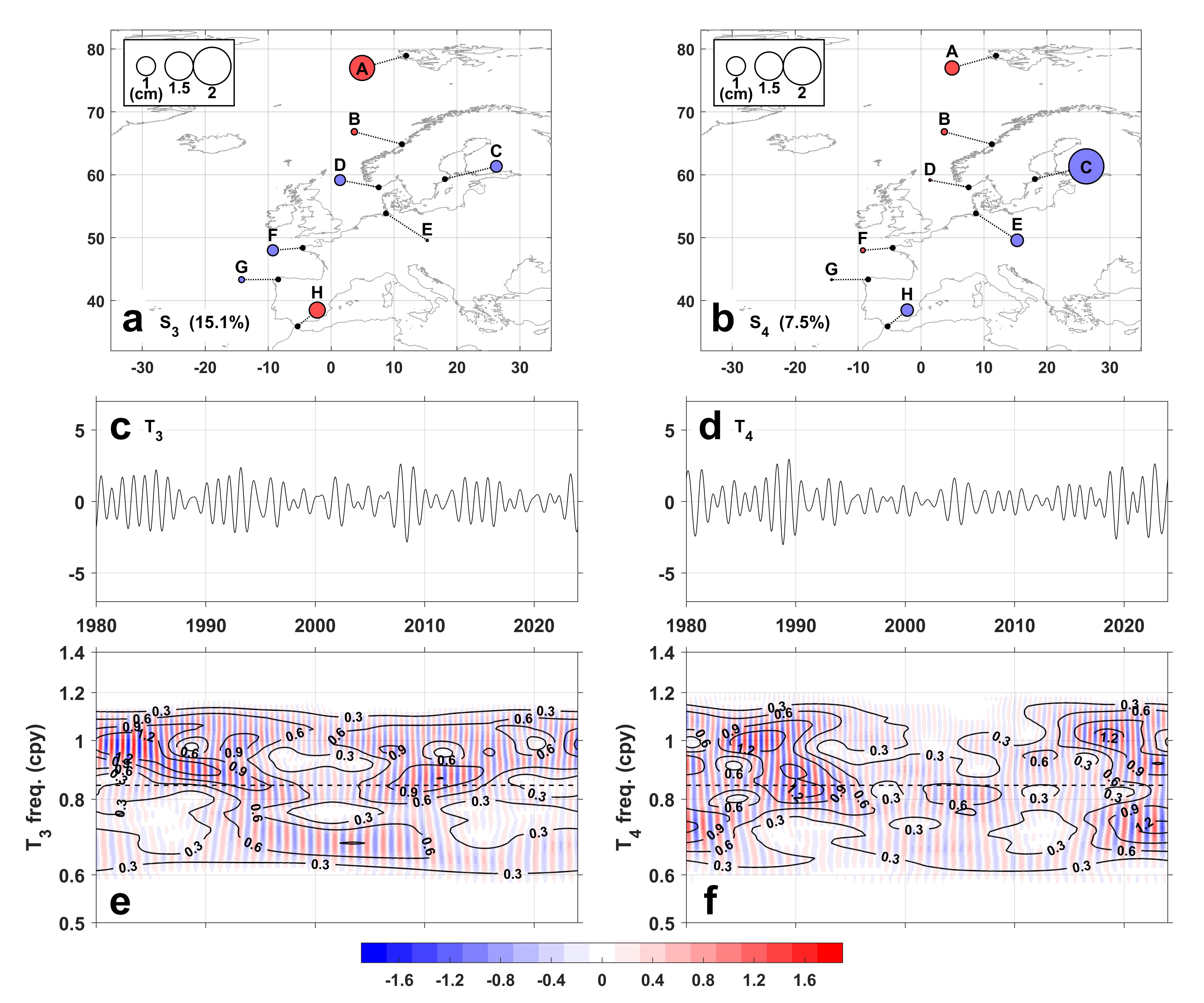


**Figure S9.** Similar to Fig. 6 in the main text, but for EOF modes 3 and 4.


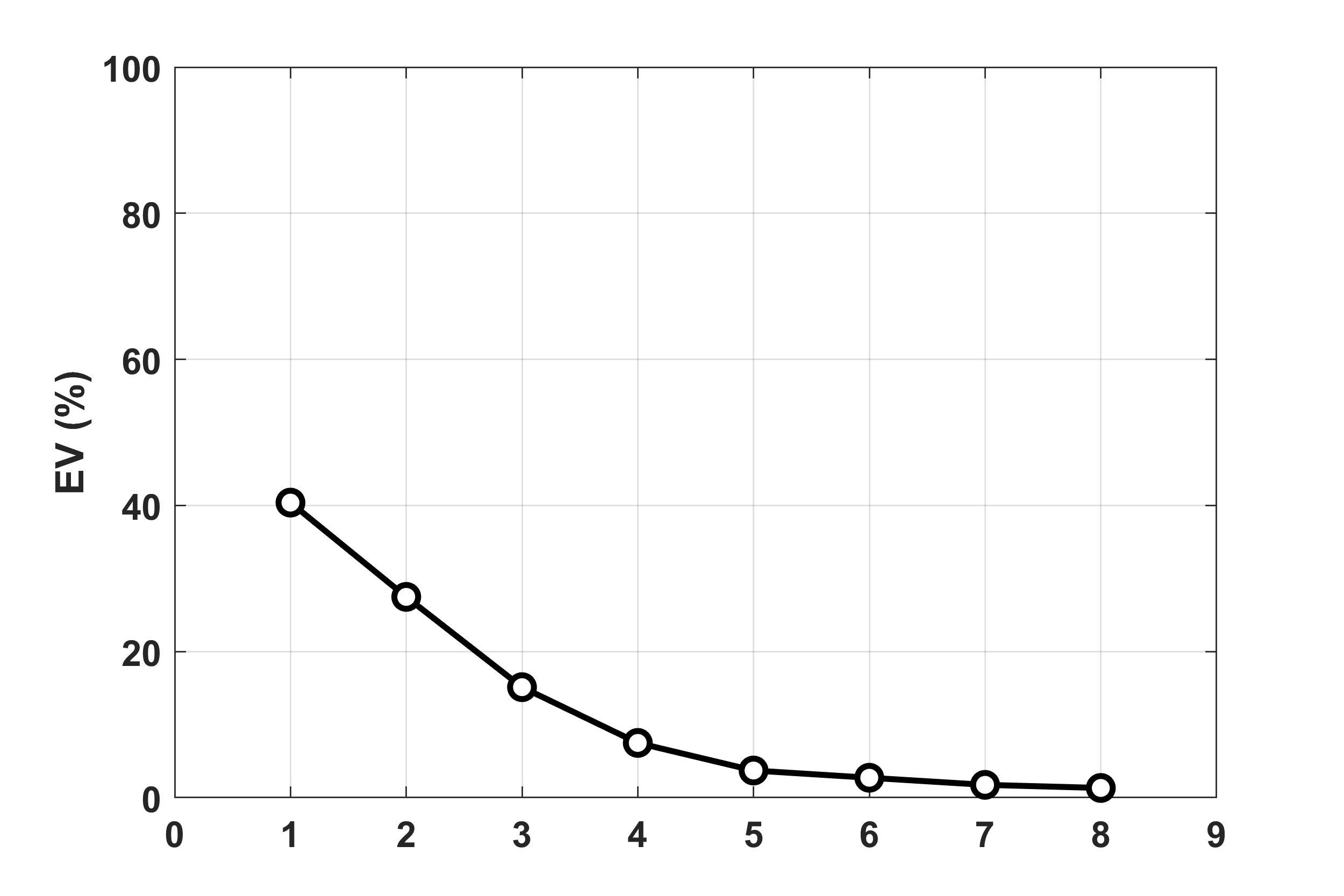


**Figure S10.** Explained variances (EV) of the EOF modes up to 8 derived from 8 stations in Europe.
